# Indoxyl Sulfate Affects Glial Function Increasing Oxidative Stress and Neuroinflammation in Chronic Kidney Disease: Interaction between Astrocytes and Microglia

**DOI:** 10.3389/fphar.2017.00370

**Published:** 2017-06-12

**Authors:** Simona Adesso, Tim Magnus, Salvatore Cuzzocrea, Michela Campolo, Björn Rissiek, Orlando Paciello, Giuseppina Autore, Aldo Pinto, Stefania Marzocco

**Affiliations:** ^1^Department of Pharmacy, University of SalernoFisciano, Italy; ^2^Ph.D. Program in Drug Discovery and Development, University of SalernoFisciano, Italy; ^3^Department of Neurology, University Medical Center Hamburg-EppendorfHamburg, Germany; ^4^Department of Biological and Environmental Sciences, University of MessinaMessina, Italy; ^5^Department of Veterinary Medicine and Animal Production, University of Naples “Federico II”Naples, Italy

**Keywords:** indoxyl sulfate, neuroinflammation, oxidative stress, neurodegeneration, uremic toxins, chronic kidney disease

## Abstract

Indoxyl sulfate (IS) is a protein-bound uremic toxin resulting from the metabolism of dietary tryptophan which accumulates in patients with impaired renal function, such as chronic kidney disease (CKD). IS is a well-known nephrovascular toxin but little is known about its effects on central nervous system (CNS) cells. Considering the growing interest in the field of CNS comorbidities in CKD, we studied the effect of IS on CNS cells. IS (15–60 μM) treatment in C6 astrocyte cells increased reactive oxygen species release and decreased nuclear factor (erythroid-derived 2)-like 2 (Nrf2) activation, and heme oxygenase-1 (HO-1) and NAD(P)H dehydrogenase quinone 1 expression. Moreover, IS increased Aryl hydrocarbon Receptor (AhR) and Nuclear Factor-kB (NF-kB) activation in these cells. Similiar observations were made in primary mouse astrocytes and mixed glial cells. Inducible nitric oxide synthase and cyclooxygenase-2 (COX-2) expression, tumor necrosis factor-α and interleukin-6 release and nitrotyrosine formation were increased by IS (15–60 μM) in primary mouse astrocytes and mixed glial cells. IS increased AhR and NF-kB nuclear translocation and reduced Nrf2 translocation and HO-1 expression in primary glial cells. In addition, IS induced cell death in neurons in a dose dependent fashion. Injection of IS (800 mg/kg, i.p.) into mice induced histological changes and increased COX-2 expression and nitrotyrosine formation in thebrain tissue. Taken together, our results show a significant contribution of IS in generating a neurotoxic enviroment and it could also have a potential role in neurodegeneration. IS could be considered also a potential therapeutical target for CKD-associated neurodegenerative complications.

## Introduction

Neurodegenerative diseases have become a growing health burden and, in our aging population, are often linked to other comorbidities. Oxidative stress and neuroinflammation contribute to the pathogenesis of neuronal degeneration (Guo et al., 2002) and can cause cell membrane damage from lipid peroxidation, changes in protein structure and function, due to protein oxidation, and structural DNA damage, hallmarks of several neurodegenerative diseases (Adams and Odunze, 1991; Smith et al., 1994; Petersén et al., 1999; Frank-Cannon et al., 2009). The central nervous system (CNS) is particularly sensitive to oxidative stress, probably because of its high oxygen demand and the presence of polyunsaturated fatty acids and low levels of glutathione (GSH; Richardson et al., 1990; Roger et al., 1997). Increasing reactive oxygen species (ROS) production can exacerbate the expression of inflammatory mediators as detected in patients with neurodegenerative diseases (Hsieh and Yang, 2013).

Chronic kidney disease (CKD) is characterized by a progressive loss of renal function that, in its terminal phase, shows signs and symptoms of uremic syndrome (Vanholder et al., 2001). Patients with CKD have many comorbidities such as immune disorders, with the coexistence of immunodeficiency and immune activation, and neurological complications that largely contribute to the morbidity and mortality of this disease (Buchman et al., 2009; Krishnan and Kiernan, 2009; Marzocco et al., 2010). CKD is frequently associated with cognitive impairment and, among patients with terminal CKD receiving haemodialysis, more than 85% have cognitive deficits (Krishnan and Kiernan, 2009). Cognitive impairment in CKD is also associated with a poorer clinical outcomes (Sehgal et al., 1997; Kimmel et al., 1998; Murray and Knopman, 2010; Radic et al., 2010). Patients with CKD are also at higher risk of cognitive decline and even dementia (Seliger et al., 2004; Wang et al., 2010). Causes of cognitive impairment in CKD are multifactorial and they include cerebrovascular disease, renal anemia, secondary hyperparathyroidism, dialysis disequilibrium, and uremic toxins accumulation. Plasmatic levels of uremic toxins increase as CKD progresses, and they are believed to be the main cause of cognitive impairment (Krishnan and Kiernan, 2009). However, the exact role or mechanism of uremic toxins in cognitive disorders has not been determined yet. One of the most important uremic toxins is indoxyl sulfate (IS), a protein-bound uremic toxin, which is not effectively eliminated by dialysis. IS is a nephro-vascular toxin (Niwa, 2010) that causes nephrotoxicity especially on tubular cells, inhibits proliferation of endothelial cells and is an inducer of free radicals (Dou et al., 2007). Moreover, it has been reported that IS enhances inflammatory response and ROS in LPS-stimulated macrophages (Adesso et al., 2013). Among various uremic toxins, IS is a likely candidate capable to trigger cerebral dysfunction in kidney disease (Watanabe et al., 2014). Therefore, we chose to investigate the effects of IS on glial cells and the impact on neuronal survival, all primary aspects involved in CNS homeostasis.

## Materials and Methods

### Reagents

All reagents and compounds, unless stated otherwise were purchased from Sigma Chemicals Company (Sigma, Milan, Italy).

### Cell Culture

#### *In Vitro* Studies

C6 glioma cell line was obtained from American Type Culture Collection (ATCC; Manassas, VA, United States). C6 were cultured in DMEM, 10% FBS (mL/L), penicillin/streptomycin (100 units/0.1 mg/mL) and 2 mML-glutamine at 37°C in 5% CO_2_ atmosphere and passaged at confluence using a solution of 0.025% trypsin and 0.01% EDTA. This cell line was originally derived from rat brain tumors and have oligodendrocytic, astrocytic and neuronal properties (Benda et al., 1968; Parker et al., 1980). C6 cells are widely used as an astrocyte-like cell line (Quincozes-Santos et al., 2009).

#### *Ex Vivo* Studies: Primary Astrocytes, Microglia and Neurons

Cultures of mixed glial cell from cortex were prepared from postnatal days 1–2 mouse pups (Female C57BL/6 mice; Harlan Laboratories, Udine, Italy). Mice were housed under specific pathogen-free conditions and fed with standard chow diet at the University of Messina, Department of Chemical, Biological, Pharmaceutical and Environmental Sciences. The animal experiments were performed according protocols following the Italian and European Community Council for Animal Care (DL. 26/2014). Cerebral cortices were excised, meninges, olfactory bulb and thalami removed, and the hemispheres were transferred to petri dishes containing HBSS and were cut into four small pieces. Brains were centrifuged for 1 min at 200–300 g. The supernatant was removed and the pellet was incubated with HBSS/10 mM HEPES buffer, 0.5 mg/ml Papain, 10 μg DNAse solution for 25 min at 37°C. The extracted cells were centrifuged for 5 min at 200–300 g and the pellet was resuspend in BME medium (10% FBS and 0.5% penicillin/streptomycin). The cell suspension was filtered through a 70-μm cell strainer to remove debris. The extracted cells were suspended in BME medium (10% FBS and 0.5% penicillin/streptomycin) in 75 cm^3^ flasks. The medium was changed after 48 h and then twice per week (Gelderblom et al., 2012). After 20 days, in some flasks, to obtain only astrocytes in the culture, microglia were dislodged using an orbital shaker (200 rpm for 1 h, 37°C). Moreover, in order to further remove residual microglia from the remaining cell monolayers, it was used a 60-min exposure (50 mM) to the lysosomotropic agent Leu-Leu-OMe (<5% microglia, referred to some microglial cells not dethached from the treatments, was deteced by flow cytometry using anti-Iba1 as antibody; Marinelli et al., 2015).

Dissociated cell cultures of mouse hippocampus and cortex were established from day 16 C57B/6J mouse embryos, as previously described (Fann et al., 2013). Hippocampal and cortical neurons were plated in 35, 60, or 100-mm diameter polyethylenimine-coated plastic dishes. Primary neurons were maintained in Neurobasal medium containing 25 mM of glucose, B-27 supplement (Invitrogen), 0.001% gentamycin sulfate, 2 mML-glutamine, and 1 mM HEPES (pH 7.2) at in 5% CO_2_ atmosphere 37°C. Approximately 95% of the cells in such cultures were neurons and the remaining cells were astrocytes.

#### Cell Treatment

C6 cells and primary astrocytes and mixed glial cell cultures were plated 24 h before the experiments. The cellular medium was then replaced with fresh medium and cells were treated with IS (15–60 μM) for 24 h in all experiments, except for NF-kB and Nrf2 evaluation and AhR activation, where IS was added to cells for 20 min and 1 h, respectively.

Primary hippocampal and cortical neuronal cultures were plated for 2 weeks before the experiments. Then the cells were treated with IS (15–60 μM) for 24 h. For the experiments, we considered the list of uremic toxins provided by the European Uremic Toxin Work group (Vanholder et al., 2003) and thus used the IS concentration range found in the cerebrospinal fluid of CKD patients (Hosoya and Tachikawa, 2011).

### Measurement of ROS

Reactive oxygen species production was evaluated by the probe H_2_DCF-DA as previously reported (Pepe et al., 2015). H_2_DCF, in presence of ROS, is rapidly oxidized to the highly fluorescent DCF. C6 (3.0 × 10^5^ cells/well) and primary astrocytes and mixed glial cell cultures (1.5 × 10^5^ cells/well) were plated into 24-well plates and then IS (15–60 μM) was added. After 24 h cells were collected, washed with PBS and incubated in PBS containing H_2_DCF-DA (10 μM) at 37°C. Cellular fluorescence was evaluated using fluorescence-activated cell sorting analysis (FACSscan; Becton Dickinson) and elaborated with Cell Quest software. In some experiments, in C6 cells, either DPI (10 μM), that has frequently been used to inhibit ROS production mediated by flavoenzymes, or NAC (2 mM), a free radicals scavenger as well as a major contributor to maintenance of the cellular GSH, were added 1 h before IS. In other experiments, in C6 cells, PDTC (200 μM) or CH-223191 (1 μM), a ligand-selective antagonist of the AhR, were added 1 h before IS.

### Immunofluorescence Analysis with Confocal Microscopy

For immunofluorescence assay, C6 cells (3.0 × 10^5^/well), primary astrocytes and mixed glial cells (2.0 × 10^5^/well) were seeded on coverslips in 12-well plate and treated for 1 h with IS (30 μM). In some experiments with C6 cells, DPI (10 μM) and NAC (2 mM) were added 1 h before IS. In other experiments, CH-223191 (1 μM), was added 1 h before IS to C6 cells. Then cells were fixed with 4% paraformaldehyde in PBS and permeabilized with 0.1% Triton X-100 in PBS. After blocking with BSA and PBS, cells were incubated with rabbit anti-Nrf2 antibody (Santa Cruz Biotechnologies; sc-722; used at diluition 1:250), with mouse anti-AhR antibody (Abcam; ab2769; used at diluition 1:250) or with rabbit anti-p65 antibody (Santa Cruz Biotechnologies; sc-372; used at diluition 1:250). The slides were then washed with PBS for three times and fluorescein-conjugated secondary antibody (Immuno Reagents; used at diluition 1:2000) was added for 1 h. DAPI was used for counterstaining of nuclei. Coverslips were finally mounted in mounting medium and fluorescence images were caught using the Laser Confocal Microscope (Leica TCS SP5) as previously reported (Del Regno et al., 2015).

### HO-1, NQO1, SOD, iNOS, COX-2, and Nitrotyrosine Detection by Cytofluorimetry

C6 cells (5.0 × 10^4^/well), primary astrocytes and mixed glial cells (3.5 × 10^4^/well) were seeded on 96-well plate and treated, after 24 h, with IS (15–60 μM). After 24 h cells were collected, washed with PBS and then incubated in Fixing Solution for 20 min and then incubated in Fix Perm Solution for 30 min, at 4°C. Anti-HO-1 antibody (Santa Cruz Biotechnologies; sc-10789; 1:100), NQO1 antibody (Santa Cruz Biotechnologies; sc-376023; 1:100), superoxide dismutase (SOD) antibody (Santa Cruz Biotechnologies; sc-11407; 1:100), anti-iNOS (BD Transducion Laboratories; 610431; 1:100) antibody, anti-COX-2 (BD Transducion Laboratories; 610203; 1:100) antibody and anti-nitrotyrosine antibody (Millipore; 06-284; 1:100) were added to C6 cells, primary astrocytes and mixed glial cells. The secondary antibody (Immuno Reagents; used at diluition 1:100) was added in Fix Perm Solution and cells were evaluated using a fluorescence-activated cell sorting (FACSscan; Becton Dickinson) and elaborated with Cell Quest software as previously reported (Adesso et al., 2013).

### TNF-α and IL-6 Determination

Tumor necrosis factor-α and IL-6 concentration in the supernatant of cultured primary astrocytes and mixed glial cells stimulated for 24 h with IS (15–60 μM) were performed by an ELISA assay. For this we used commercially available kits for murine TNF-α and IL-6 (e-Biosciences, San Jose, CA, United States) as previously reported (Marzocco et al., 2015).

### Cytotoxicity Assay on Primary Cortical and Hippocampal Neuronal Cultures

The cytotoxic potential of IS (15–60 μM) on primary neuronal cultures after 3 h of treatment was performed using the Cytotoxicity Detection KitPLUS LDH (Roche) according to the manufacturer’s instructions. This assay was based on the evaluation of LDH activity. In the evaluation three controls are included: the first was the background control (assay medium), the second was low control (untreated cells), and the last was the high control (maximum LDH release). To determine the experimental absorbance values, the average absorbance values of the samples and controls were calculated and subtracted from the absorbance values of the background control. The percentage of cytotoxicity was determined using the equation:

$$\begin{array}{cc} \text{Cytotoxicity}(\%)= & \left(\text{exp. value}-\text{low control}\right)/\\ & \left(\text{high control}-\text{low control}\right)\times 100. \end{array}$$

### *In Vivo* Studies

Female C57BL/6 mice (6–8 weeks; Harlan Laboratories, Udine, Italy) were fed a standard chow diet and housed under specific pathogen-free conditions at the University of Messina Animal Care Review Board approved the study. The animal experiments were performed following the regulations in Italy (D.M. 116192), Europe (O.J. of E.C. L 358/1 12/18/1986), United States (Animal Welfare Assurance No. A5594-01, Department of Health and Human Services, United States).

IS was dissolved in PBS and it was injected into mice (800 mg/kg, i.p. given once) (Ichii et al., 2014). After 3 h of treatment, animals were sacrified and kidneys, brains and serum were collected and stored for the analysis.

### IS Serum Evaluation by HPLC

IS levels in mice serum were evaluated according the methods of Zhu et al. (2011) as previously reported (Marzocco et al., 2013).

### Serum Nitrite/Nitrate, TNF-α, and IL-6 Evaluation

Nitrite/nitrate, TNF-α, IL-6 release was evaluated on serum samples of mice treated with IS (800 mg/kg) for 3 h. Serum nitrite/nitrate (NOx) concentration is a marker of NO levels. For the evaluation, serum samples were incubated with FAD (50 μm), NADPH (1 mm), and nitrate reductase (0.1 U/mL). The samples were then incubated with sodium pyruvate (10 mm) and LDH (100 U/mL) for 5 min. The total NOx concentration was measured by Griess reaction adding 100 μL of Griess reagent (0.1% naphthylethylenediamine dihydrochloride in H_2_O and 1% sulfanilamide in 5% conc. H_2_PO_4_; 1:1 v/v) to 100 μL of serum treated samples, each in triplicate. The optical density at 550 nm (OD550) was measured at 540 nm in a microplate reader Titertek (Dasit, Cornaredo, Milan, Italy) and the NOx concentrations (μM) in the samples were calculated from a standard curve of sodium nitrite (Bianco et al., 2012).

TNF-α and IL-6 concentration in serum mice was assessed by an ELISA (e-Biosciences, San Jose, CA, United States).

### Histology and Immunohistochemistry

For the histological examination, kidney and brain from sacrificed mice were immediately incised and fixed in 10% formalin. For the morphological evaluation paraffin-embedded 4 μm sections were stained with haematoxylin and eosin (H&E). For the immunohistochemistry analysis, 4-μm-thick sections of the brain and kidney tissue were collected on silane-coated glass slides (Bio-Optica, Milan, Italy). Immunohistochemical stain was performed using HRP conjugated antibodies. Antigen retrieval pretreatments were performed using a HIER citrate buffer pH 6.0 (Bio-Optica, Milan, Italy) for 20 min at 98°C. EP activity was quenched with 3% H_2_O_2_ in methanol and sections were treated with a blocking solution (MACH1, Biocare Medical LLC, Concord, CA, United States) for 30 min each. Slides were then incubated overnight at 4°C with primary antibody diluted in PBS (0.01 M PBS, pH 7,2).

The primary antibodies used were: a mouse anti-COX-2 [BD Transduction Laboratories used at dilution 1:250; a rabbit anti-nitrotyrosine purchased from Millipore (Temecula, CA) used at dilution 1:100].

Antigen-antibody binding was detected by a HRP polymer detection kit (MACH1, Biocare Medical LLC, Concord, CA, United States). Antibody deposition was visualized using the DAB chromogen diluted in DAB substrate buffer and the slides were counterstained with haematoxylin. Between all incubation steps, slides were washed two times (5 min each) in PBS. For each tissue section, a negative control was performed using an irrelevant mouse or rabbit Ab.

### Data Analysis

Data are presented as standard error of the mean (SEM) showing the combined data of at least three independent experiments each in triplicate. Statistical analysis was performed by analysis of variance test, and multiple comparisons were made by Bonferroni’s test. A *P*-value lower than 0.05 was considered significant.

## Results

### IS Enhanced ROS Release in C6 Cells

**FIGURE 1:**
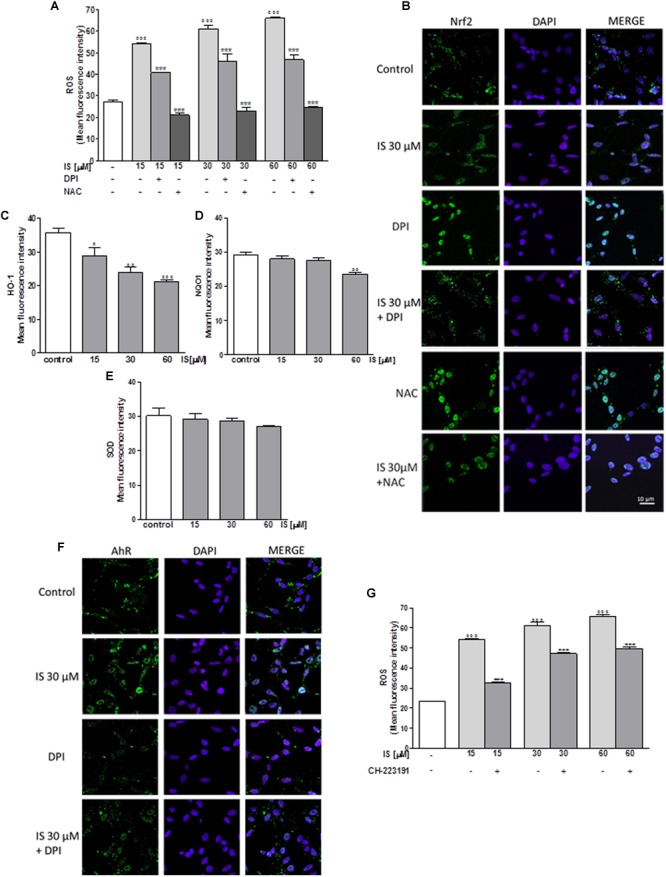
Effect of IS (15–60 μM) on ROS formation **(A)**, evaluated by means of the probe H_2_DCF-DA, in C6 cells in presence of DPI and of NAC. Cellular fluorescence was evaluated using fluorescence-activated cell sorting analysis (FACSscan; Becton Dickinson) and elaborated with Cell Quest software. Values are expressed as mean fluorescence intensity (*n* = 12). Effect of IS (30 μM) on Nrf2 nuclear translocation in C6 cells in presence of DPI and NAC **(B)**. Nuclear translocation of Nrf2 was detected using immunofluorescence confocal microscopy. Scale bar, 10 μm. Blue and green fluorescences indicate localization of the nucleus (DAPI) and Nrf2, respectively. Analysis was performed by confocal laser scanning microscopy. Effect of IS (15–60 μM) on HO-1 **(C)**, NQO1 **(D)**, and SOD **(E)** expression in C6 cells. Cellular fluorescence was evaluated using fluorescence-activated cell sorting analysis (FACSscan; Becton Dickinson) and elaborated with Cell Quest software. Effect of IS (30 μM) on AhR nuclear translocation in presence of DPI in C6 cells **(F)**. Nuclear translocation of AhR was detected using immunofluorescence confocal microscopy. Scale bar, 10 μm. Blue and green fluorescences indicate localization of nucleus (DAPI) and AhR, respectively. Analysis was performed by confocal laser scanning microscopy and values are expressed as mean fluorescence intensity (*n* = 12). Effect of IS (15–60 μM) on ROS formation **(G)**, evaluated by means of the probe H_2_DCF-DA, in C6 cells in presence of CH-223191. Values are expressed as mean fluorescence intensity (*n* = 9). ^∘∘∘^, ^∘∘^, and °denote *P* < 0.001, *P* < 0.01, and *P* < 0.05 vs. control. ^∗∗∗^ denotes *P* < 0.001, vs. IS alone.

In order to assess the effect of IS on oxidative stress in C6 cells, we evaluated intracellular ROS production. Our results indicated that IS at all tested concentrations (15–60 μM), induced a significant and concentration-dependent increase in ROS production (*P* < 0.001 vs. control; **Figure 1A**). We examined ROS production also in presence of DPI (10 μM) and NAC (2 mM). As shown in **Figure 1A**, DPI and NAC significantly inhibited ROS release induced by IS (*P* < 0.001 vs. IS alone, **Figure 1A**).

### IS Reduced Nrf2 Nuclear Translocation in C6 Cells

Following its activation, Nrf2 translocates into the nucleus and regulates cell protective gene expression. We labeled Nrf2 with a green fluorescence to track the influence of IS (30 μM) added for 1 h. In presence of IS, we observed a reduction in Nrf2 nuclear translocation (**Figure 1B**). To study the mechanisms of IS-induced reduction, we also examined Nrf2 nuclear translocation after treatment with IS (15–60 μM) in the presence of DPI and NAC. As shown in **Figure 1B**, NAC more than DPI increased Nrf2 nuclear translocation compared to IS alone (*P* < 0.01 vs. IS, **Figure 1B**).

### IS Reduced HO-1, NQO1, and SOD Expression in C6 Cells

Enzymes dealing with oxygen radicals are HO-1, NQO1, and SOD. In oder to assess their expression profile in the presence of IS, we treated C6 cells with IS (15–60 μM). After 24 h, we observed a decrease in HO-1 and NQO1 expression (*P* < 0.05 vs. control for HO-1, *P* < 0.01 vs. control for NQO1; **Figures 1C,D**). A weak inhibition by IS was observed on SOD expression (**Figure 1E**).

### IS Induced AhR Activation in C6 Cells

Aryl hydrocarbon Receptor (AhR) is the believed binding partner of IS. Therefore, we investigated AhR activation, through a green fluorescent labeling, in the presence of IS (30 μM) and DPI. After 1 h nuclear presence of AhR was increased after IS treatment and the IS effect could partially be blocked by DPI (**Figure 1F**).

To evaluate the possible involvement of AhR in ROS release induced by IS, we analyzed ROS production in presence of the AhR inhibitor: CH-223191 (1 μM). CH-223191 significantly reduced IS induced ROS production (*P* < 0.001 vs. IS; **Figure 1G**).

### IS Induced p65 NF-kB Nuclear Translocation in C6 Cells

**FIGURE 2:**
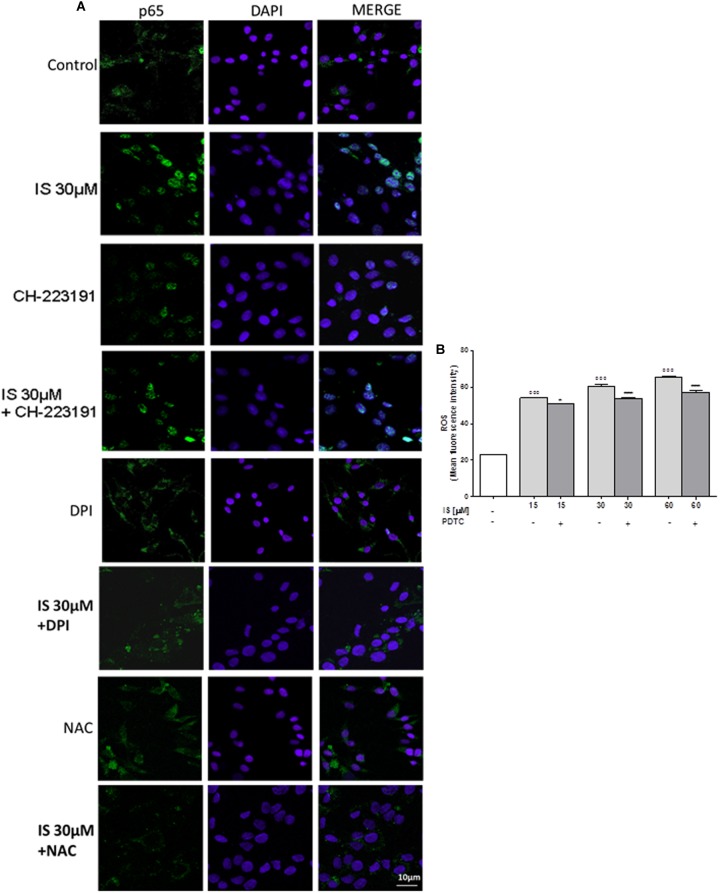
Effect of IS (30 μM) on p65 nuclear translocation in C6 cells in presence of the antagonists CH-223191, DPI and NAC in C6 cells **(A)**. Nuclear translocation of NF-kB p65 subunit was detected using immunofluorescence confocal microscopy. Scale bar, 10 μm. Blue and green fluorescences indicate localization of the nucleus (DAPI) and p65, respectively. Analysis was performed by confocal laser scanning microscopy. Effect of IS (15–60 μM) on ROS formation **(B)**, evaluated by means of the probe H_2_DCF-DA, in C6 cells in presence of NF-kB-inhibitor PDTC. Values are expressed as mean fluorescence intensity (*n* = 9).^∘∘∘^ denotes *P* < 0.001 vs. control. ^∗∗∗^ denotes *P* < 0.001 and ^∗^ denotes *P* < 0.05 vs. IS alone.

Nuclear factor-kB p65 was labeled with a green fluorescence to track the effect of IS (30 μM) on NF-kB activation. p65 nuclear traslocation resulted increased after IS treatment (**Figure 2A**).

The IS-induced p65 NF-kB nuclear translocation was inhibited by DPI and NAC and to a lesser extent by CH-223192 (**Figure 2A**). To evaluate the possible involvement of NF-kB in ROS release induced by IS, we analyzed ROS production in presence of a NF-kB inihibitor: PDTC (200 μM; **Figure 2B**). PDTC significantly reduced IS induced ROS production (*P* < 0.05 vs. IS alone; **Figure 2B**).

### IS Influenced Oxidative Estress and Pro-inflammatory Parameters in Primary Astrocytes and Mixed Glial Cell Cultures

**FIGURE 3:**
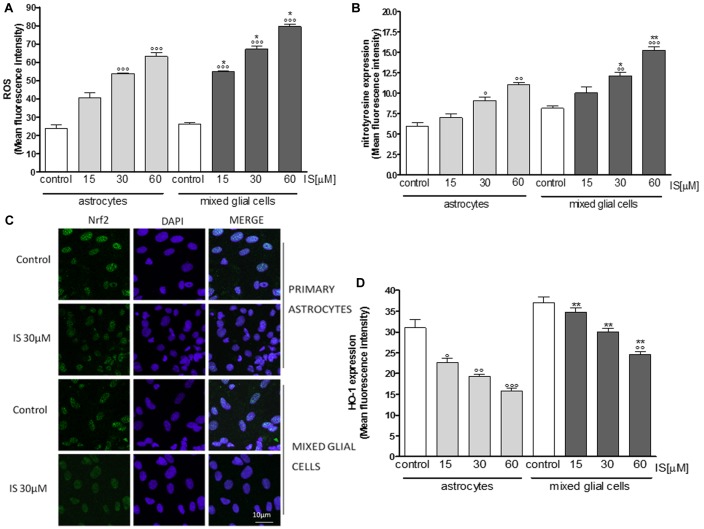
Effect of IS (15–60 μM) on ROS formation **(A)**, evaluated by means of the probe H_2_DCF-DA, in astrocytes and mixed glial cells. Cellular fluorescence was evaluated using fluorescence-activated cell sorting analysis (FACSscan; Becton Dickinson) and elaborated with Cell Quest software. Effect of IS (15–60 μM) on nitrotyrosine formmation **(B)** in astrocytes and mixed glial cells. Cellular fluorescence was evaluated using fluorescence-activated cell sorting analysis (FACSscan; Becton Dickinson) and elaborated with Cell Quest software. Effect of IS (30 μM) on Nrf2 nuclear translocation in astrocytes and mixed glial cells **(C)**. Nuclear translocation of Nrf2 was detected using immunofluorescence confocal microscopy. Scale bar, 10 μm. Blue and green fluorescences indicate localization of nucleus (DAPI) and Nrf2, respectively. Analysis was performed by confocal laser scanning microscopy. Effect of IS (15–60 μM) on HO-1 expression **(D)** in astrocytes and mixed glial cells. Cellular fluorescence was evaluated using fluorescence-activated cell sorting analysis (FACSscan; Becton Dickinson) and elaborated with Cell Quest software. Values are expressed as mean fluorescence intensity (*n* = 9). ^∘∘∘^, ^∘∘^, and °denote *P* < 0.001, *P* < 0.01, and *P* < 0.05 vs control. ^∗∗^ and ^∗^ denote *P* < 0.01 and *P* < 0.05 vs. astrocytes.

Primary astrocytes and mixed glial cell cultures are a less artificial cell culture system. In these cells IS (15–60 μM) also induced a significant ROS production (*P* < 0.001 vs. control; *P* < 0.05 vs. astrocytes alone; **Figure 3A**).

**FIGURE 4:**
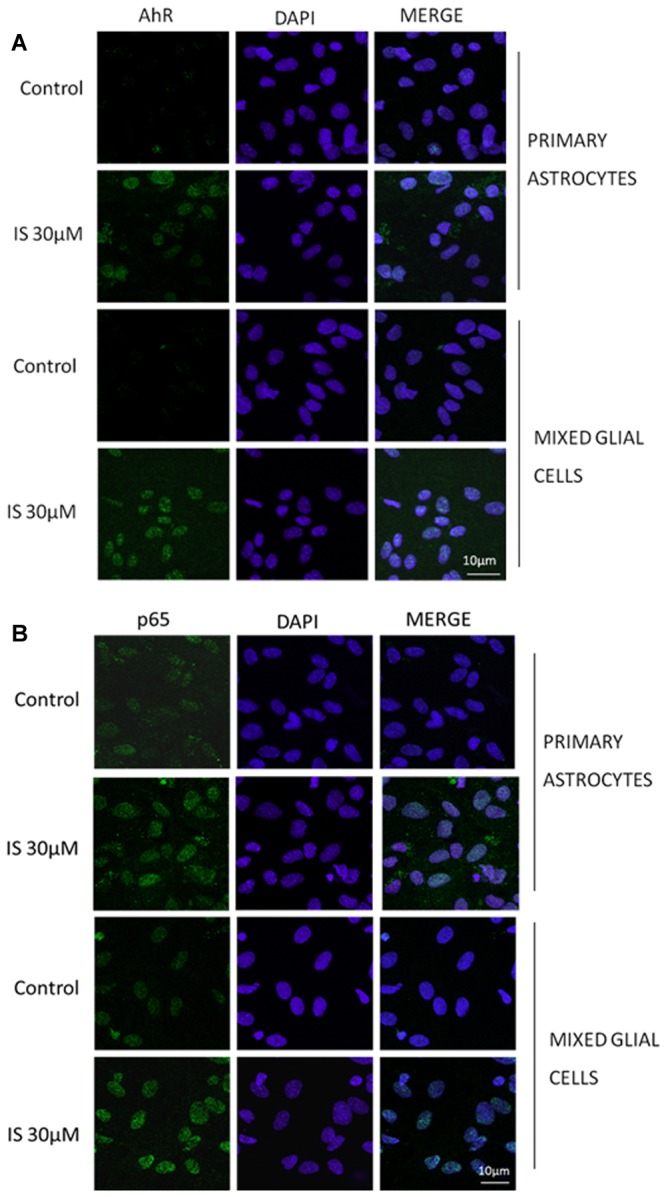
Effect of IS (30 μM) on AhR **(A)** and p65 **(B)** nuclear translocation in astrocytes and mixed glial cells. Nuclear translocation of AhR and p65 was detected using immunofluorescence confocal microscopy. Scale bar, 10 μm. Blue and green fluorescences indicate localization of nucleus (DAPI) and AhR and p65, respectively. Analysis (*n* = 9) was performed by confocal laser scanning microscopy.

IS led to an increase of nitrotyrosine formation (*P* < 0.05 vs. control, *P* < 0.05 vs. astrocytes alone; **Figure 3B**) and to a reduction of Nrf2 translocation (**Figure 3C**) and HO-1 expression (*P* < 0.05 vs. control; **Figure 3D**). In primary mixed glial cell cultures the response was much more prominent indicating the contribution of the microglial cells. Comparable to the C6 cell lines, IS (30 μM) added for 1 h enhanced nulcear translocation of AhR and p65 (**Figures 4A,B**).

**FIGURE 5:**
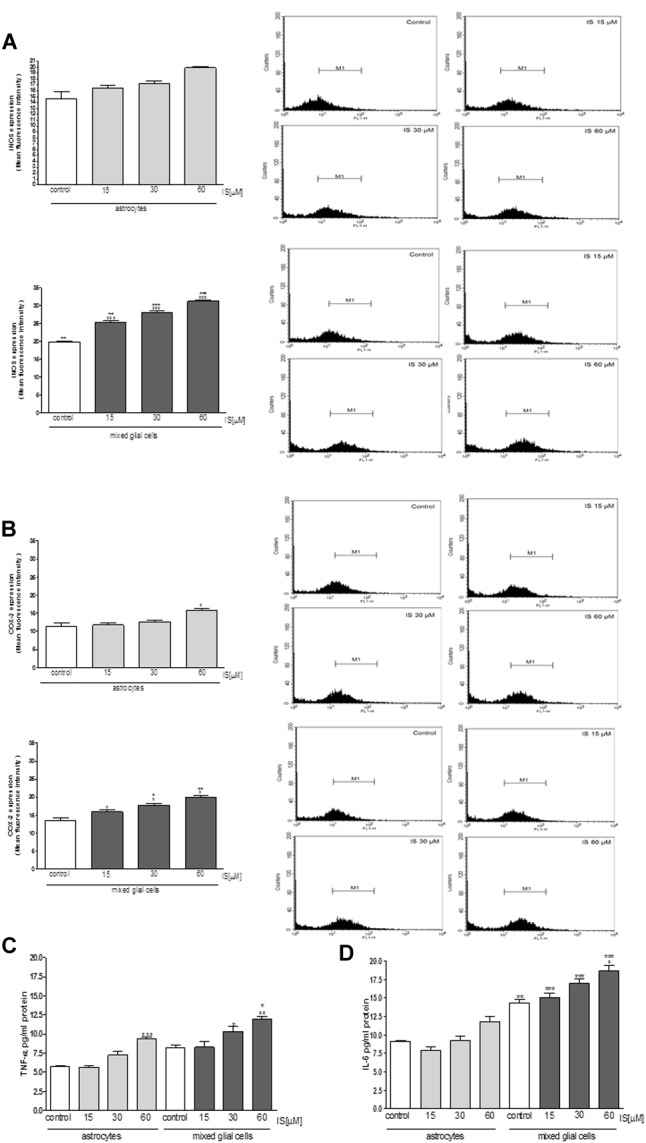
Effect of IS (15–60 μM) on iNOS **(A)**, COX-2 **(B)** expression by astrocytes and mixed glial cells. Cellular fluorescence was evaluated using fluorescence-activated cell sorting analysis (FACSscan; Becton Dickinson) and elaborated with Cell Quest software. Values are expressed as mean fluorescence intensity (*n* = 9). Effect of IS (15–60 μM) TNF-α **(C)** and IL-6 **(D)** release by astrocytes and mixed glial cells (*n* = 9). Cyokine release was assessed by ELISA assay and expressed as pg/ml (*n* = 9). ^∘∘∘^, ^∘∘^, and °denote *P* < 0.001, *P* < 0.01, and *P* < 0.05 vs. control. ^∗∗∗^,^∗∗^, and ^∗^ denote *P* < 0.001, *P* < 0.01, and *P* < 0.05 vs. astrocytes.

Moreover under the same experimental conditions, we observed a significant increase in iNOS and COX-2 expression in astrocytes and mixed glial cell cultures treated with IS (15–60 μM; *P* < 0.001 vs. control and *P* < 0.01 vs. astrocytes alone; **Figures 5A,B**). IS treatment also induced a significant production of TNF-α in astrocytes and mixed glial cell cultures and IL-6 in mixed glial cell cultures (*P* < 0.05 vs. control and *P* < 0.05 vs. astrocytes alone; **Figures 5C,D**).

### IS Increased Cellular Death in Neuronal Cultures

**FIGURE 6:**
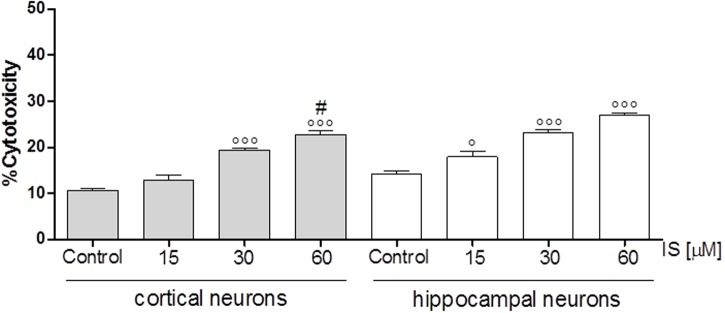
Effect of IS (15–60 μM) on cortical and on hippocampal neuronal cell viability. Values are expressed as percentage of cytotoxicity (n = 9). ^∘∘∘^and ° denote *P* < 0.001 and *P* < 0.05 vs. control, # denotes *P* < 0.05 vs. hippocampal neurons.

In order to investigate the effect of IS on neuronal death we used cortical and hippocampal neuron cultures. Our results showed that both, cortical and hippocampal neurons, are susceptible to IS-induced neuronal cell death in a dose-dependent fashion (*P* < 0.05 vs. control, *P* < 0.005 vs. hippocampal neurons; **Figure 6**).

### IS enhanced NO, TNF-α, and IL-6 Levels in Mice Serum and Increased COX-2 and Nitrotyrosine Expression in Brain and Kidney

To match our *in vitro* findings with the *in vivo* situation, we injected mice with IS (800 mg/Kg) which resulted in a significantly higher IS serum concentration (79.66 ± 1.67 μM vs. 0.55 ± 0.00 μM, *P* < 0.001 vs. control). Total nitrite serum increased significantly in IS-treated mice compared to control mice (89.76 ± 9.98 vs. 55.99 ± 9.69 μM, *P* < 0.05). TNF-α and IL-6 evaluation indicated that IS induced a weak increase in TNF-α serum levels (18.91 ± 1.77 vs. 17.43 ± 2.02 of control group; *P* = NS) but a significant increase if IL-6 serum levels (23.99 ± 3.38 vs. 12.93 ± 2.48 of control group; *P* < 0.05).

**FIGURE 7:**
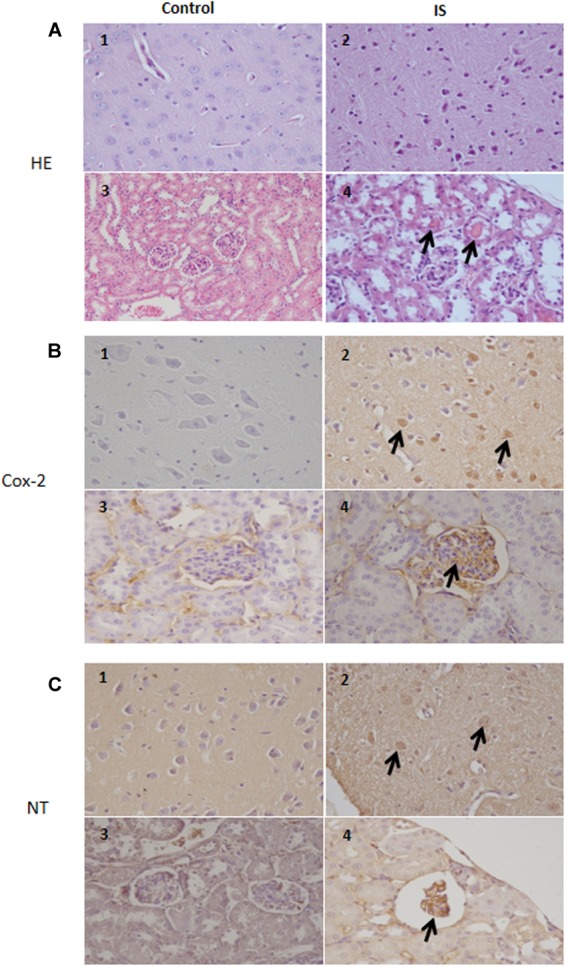
Histologic and immunohistochemical findings of brain and kidneys in treated mice (IS column). **(A)** (1) Brain; normal tissue from control mouse. (2) Brain; neuronal pyknosis associated with mild satellitosis. (3) Kidney; normal tissue from control mouse. (4) Kidney; Atrophic glomeruli and severe vacuolar degeneration of tubules with proteinaceous amorphous material and hypereosinophilic concretions within lumen (arrows); *Hematoxylin and Eosin (HE) stain*. **(B)** (1) Brain; normal tissue from control mouse. (2) Brain; strong immunoreactivity for COX-2 antibody in degenerating neurons (arrows) from treated mouse. (3) Kidney; normal tissue from control mouse. (4) Kidney; strong immunoreactivity for COX-2 antibody in blood vessels of the glomeruli (arrows) from treated mouse. *Immunohistochemistry (HRP-method)*. **(C)** (1) Brain; normal tissue from control mouse. (2) Brain; the immunoreactivity with the anti-nitrotyrosine antibody is intensely detected in the neurons of a treated mice (arrows). (3) Kidney; normal tissue from control mouse. (4) Kidney; strong immunoreactivity in blood vessels of an atrophic glomerulus (arrow) from treated mouse. *Immunohistochemistry (HRP-method)*. Data are from two independent experiments and represent mean ± SEM (*n* = 5–10 per group).

Our results indicate a COX-2 immunoreactivity in a subset of neurons in the brain tissue in normal as well as treated mice. In the treated mice, more cells showed an immunoreactivity which extended to degenerating neurons and blood vessels. Also in the kidney, we observed a strong COX-2 staining primarily in the glomeruli (**Figure 7**). Similarily, the anti-nitrotyrosine antibody stained neurons of the treated mice, while we saw only weak staining in the control group. Also in the kidney the immunostaining of the glomeruli was stronger in the treated mice compared to control (**Figure 7**).

### IS Enhanced Not Only Kidney Cell Damage but Also Neuronal Cell Damage

According to previous observations (Ichii et al., 2014), we found atrophic glomeruli with thickening of the Bowman’s capsule and mesangial matrix and aspects of segmental solidification after IS treatment (**Figure 7**). The tubular epithelial cells showed granule-fatty degeneration and sometimes vacuoles and were arranged around amorphous and hypereosinophilic protein aggregates (“casts”; **Figure 7**). We observed interstitial edema, dilatation of renal arterioles and small hemorrhagic areas (**Figure 7**).

We could also observed IS effects in the brain. Histological evaluation showed some neurons showing cytoplasm angular margins, with eosinophilic cytoplasm and pyknotic nuclei (neuronal necrosis). Around the necrotic neurons were slightly hyperplastic glial cells (satellitosis).

## Discussion

In this study, we can provide evidence that IS can directly influence glial function and can cause neuronal damage, implicating IS directly in the pathways by which CKD influences cognitive functions. Cognitive impairment of CKD patients is one of the main complications despite pharmacological and dialytic treatment (Vanholder et al., 2001; Raff et al., 2008; Di Micco et al., 2012). We were able to show that IS induces oxidative stress and inflammatory mediators in glial cells. Oxidative stress and inflammation are essential for defense against injuries, but, if not properly regulated, they are capable of initiating various deleterious effects (Libetta et al., 2011). Oxidative stress increases together with the progression of CKD and it correlates with the level of renal function (Popolo et al., 2013) and, therefore, also with IS levels. In addition, the antioxidant systems are also compromised in CKD patients and worsen with the progression of renal failure (Morena et al., 2002). Thus, the control of inflammation and oxidative stress is of particular importance in uremic syndrome.

Our observations point to specific pathways underlying the oxidative stress and inflammation induced by IS in glial cells: (i) NADPH oxidase and glutathione levels, (ii) AhR and NF-kB activation, (iii) a reduced antioxidant response Nrf2-mediated, (iv) activation of pro-inflammatory mediators, and (v) alteration in glial proliferation/cell cycle. Moreover, we find a direct link between IS and neuronal damage linking IS to neurotoxicity. We found that IS induced a significant and concentration-related ROS release from cultured C6 astrocytes, primary astrocytes and to an even greater extent in mixed glial cell culture. Mechanistic studies revealed that both NADPH oxidase, as evaluated by the presence of DPI, and GSH homeostasis, as evaluated by NAC addition, are involved in IS-induced ROS release. These results are in accordance with previous studies reporting that IS interfered both with pro- and anti-oxidant factors in endothelial cells (Dou et al., 2007; Yu et al., 2011a), vascular smooth muscle cells (Mozar et al., 2011), kidney cells (Shimizu et al., 2013), and macrophages (Adesso et al., 2013). It has been also reported that NAD(P)H oxidase levels increased in CKD patients and in experimental models of renal insufficiency (Fortuno et al., 2005; Castilla et al., 2008). IS is a potent AhR ligand (Schroeder et al., 2010). In the brain, AhR is ubiquitously expressed including the cerebral cortex, hippocampus, and cerebellum (Lin et al., 2008). It has been implicated in sensorimotor and cognitive dysfunctions caused by oxidative stress or excitotoxicity (Kim and Yang, 2005; Williamson et al., 2005; Lin et al., 2008). Our results indicated that IS activates AhR in astrocytes which likely promoted further oxidative stress. This result fits with data reporting an AhR-mediated oxidative stress pathway in human vascular endothelial cells (Watanabe et al., 2013). Interestingly, our data gives further insight in IS-induced AhR-ROS pathway in astrocytes since treatment with IS in presence of DPI is able to reduce AhR activation. Here we report that IS also activated NF-κB and previous studies indicated a reciprocal interaction between NF-κB and Nrf2 (Bolati et al., 2013). IS-induced ROS is able to induce NF-kB activation. This activation could, in turn, be responsible for the Nrf2 downregulation (Bolati et al., 2013), because the interaction of p65 with Keap1 promotes reduction of Nrf2 protein level through Nrf2 ubiquitination (Yu et al., 2011b). Moreover, the upregulation of p53 expression induced by IS-induced NF-κB activation is involved in the suppression of Nrf2 mRNA expression (Faraonio et al., 2006). Our data indicate a cross-talk between ROS and NF-kB because, DPI and NAC treatment were able to reduce NF-kB activation and NF-kB inhibition was able to interfere with ROS release in astrocytes. Nrf-2 is a transcription factor responsible for the regulation of the cellular redox balance and protective antioxidant and phase II enzymes (Kensler et al., 2007). Nrf2 binding to the antioxidant response element (ARE) induced the regulation of some anti-oxidant proteins such as HO-1 and NQO1 (Kansanen et al., 2013). We found that IS also reduced HO-1 and NQO1 expression, thus further contributing to a decrease of antioxidant defenses and to oxidative stress-induced damage in CNS cells.

Astrocytes are the most abundant glial cells in the CNS and they have a number of important physiological properties related to the homeostatic control of the extracellular environment. Astrocyte cells provide structural, trophic, and metabolic support to neurons, modulate synaptic activity and are involved in multiple brain functions contributing to neuronal development. Moreover, astrocytes actively participate in processes triggered by brain injuries, aimed at repairing brain damage (Vernadakis, 1996; Marchetti, 1997). It has been recently reported that astrocytes contribute actively to various forms of dementia (Rodríguez et al., 2009) and disturbances in the complex neuron–glia interaction are increasingly recognized as an important pathophysiological mechanism in a wide variety of neurological disorders including neurodegeneration (Erol, 2010).

In response to a variety of stimuli and pathological events, astrocytes and microglia become activated. Microglia, activated earlier than astrocytes, promotes astrocytic activation by releasing inflammatory mediators and ROS. On the other hand, activated astrocytes facilitate the activation of distant microglia, and in some cases also inhibit microglial activities (Tremblay et al., 2011; Kingwell, 2012; Liu et al., 2012). Thus, atrocytes–microglia interactions are important in regulating both physiological and pathological conditions. We could demonstrate that in mixed glial cell cultures stimulation with IS resulted in higher levels of ROS and proinflammatory mediators.

Activated microglia and astrocytes also release a variety of cytokines, chemokines, and toxic factors, such as TNF-α, IL-6, and NO, all of which may lead to neuronal toxicity and result in the aggressive neuronal apoptosis, that has been reported as the most crucial event in neuronal loss of neurological diseases (D’Amelio et al., 2010; Allaman et al., 2011; Heneka et al., 2015; Varley et al., 2015). In this study, we observed that IS significant increases in astrocytes and mixed glial cells cytokines production and of pro-inflammatory enzymes as iNOS and COX-2 that, together with oxidative stress conditions can influence neuronal death, involved in neurodegeneration. Moreover, we observed that IS increased neuronal cell death in cortical and hippocampal neurons thus supporting its effect in neuronal loss.

Taken together, we can show that the AhR is important for the IS induced activation of NF-kB, ROS and pro-inflammatory cytokine production, and downregulation of cell protective factors such as Nrf2, HO-1 or NQO1 in glial cells. Some of these pathways can be specifically blocked. Evidences of IS-induced effects on CNS are here supported also by *in vivo* experiments. IS induced histological brain alterations and the expression of oxidative stress and inflammatory markers, such as nitrotyrosine and COX-2. Until now, there was little information about the potential of IS on SNC cells. Taken together, our results highlight the effect of IS on CNS homeostasis. This study adds to hypothesis that IS significantly contributes to neurological complications observed in CKD, and that its levels could be not only a marker of disease progression but also a pharmacological target in cognitive dysfunction observed in CKD.

## Author Contributions

SA drafted the manuscript, participated in research design and carried out the experiments; TM contributed to conceive and design the experiments and to the writing of the manuscript; SC and BR contributed to the writing of the manuscript and designing the experiments; MC participated to the *in vivo* experiments; OP performed the histological and immunohistochemical analysis; GA contributed to analyze the data; AP contributed to data analysis and to the writing of the manuscript; SM conceived and designed the research, the experiments and contributed to the writing of the manuscript. All authors read and approved the final manuscript.

## Conflict of Interest Statement

The authors declare that the research was conducted in the absence of any commercial or financial relationships that could be construed as a potential conflict of interest. The reviewer DC and handling Editor declared their shared affiliation, and the handling Editor states that the process nevertheless met the standards of a fair and objective review.

## Abbreviations

AhR: Aryl hydrocarbon Receptor
CKD: chronic kidney disease
CNS: central nervous system
COX-2: cyclooxygenase-2
DCF: 2′,7′-dichlorofluorescein
DPI: diphenyleneiodonium
ELISA: enzyme-linked immuno sorbent assay
EP: endogenous peroxidase
H_2_DCF-DA: 2′,7′-dichlorofluorescin-diacetate
H_2_O_2_: hydrogen peroxide.
HO-1: heme oxygenase-1
HRP: horseradish peroxidase
IL-6: interleukin-6
iNOS: inducible nitric oxide synthase
IS: indoxyl sulfate
LDH: lactate dehydrogenase
NAC: *N*-acetylcysteine
NF-kB: nuclear factor-kB
NQO1: NAD(P)H dehydrogenase quinone 1
Nrf2: nuclear factor (erythroid-derived 2)-like 2
PBS: phosphate buffer saline
PDTC: pyrrolidine dithiocarbamate
ROS: reactive oxygen species
TNF-α: tumor necrosis factor-α.

## References

[B1] Adams J. D. Jr. Odunze I. N. (1991). Oxygen free radicals and Parkinson’s disease. *Free Radic. Biol. Med.* 10 161–169. 10.1016/0891-5849(91)90009-R 2016074

[B2] Adesso S. Popolo A. Bianco G. Sorrentino R. Pinto A. Autore G. (2013). The uremic toxin indoxyl sulphate enhances macrophage response to LPS. *PLoS ONE* 8:e76778 10.1371/journal.pone.0076778 PMC3786936 24098806

[B3] Allaman I. Belanger M. Magistretti P. J. (2011). Astrocyte-neuron metabolic relationships: for better and for worse. *Trends Neurosci.* 34 76–87. 10.1016/j.tins.2010.12.001 21236501

[B4] Benda P. Lightbody J. Sato G. Levine L. Sweet W. (1968). Differentiated rat glial cell strain in tissue culture. *Science* 161 370–371. 10.1126/science.161.3839.370 4873531

[B5] Bianco G. Russo R. Marzocco S. Velotto S. Autore G. Severino L. (2012). Modulation of macrophage activity by aflatoxins B1 and B2 and their metabolites aflatoxins M1 and M2. *Toxicon* 59 644–650. 10.1016/j.toxicon.2012.02.010 22402176

[B6] Bolati D. Shimizu H. Yisireyili M. Nishijima F. Niwa T. (2013). Indoxyl sulfate, a uremic toxin, downregulates renal expression of Nrf2 through activation of NF-κB. *BMC Nephrol.* 14:56 10.1186/1471-2369-14-56 PMC3599003 23496811

[B7] Buchman A. S. Tanne D. Boyle P. A. Shah R. C. Leurgans S. E. Bennett D. A. (2009). Kidney function is associated with the rate of cognitive decline in the elderly. *Neurology* 73 920–927. 10.1212/WNL.0b013e3181b72629 19657107 PMC2754333

[B8] Castilla P. Davalos A. Teruel J. L. Cerrato F. Fernandez-Lucs M. Merino J. L. (2008). Comparative effects of dietary supplementation with red grape juice and vitamin E on production of superoxide by circulating neutrophil NADPH oxidase in hemodialysis patients. *Am. J. Clin. Nutr.* 87 1053–1061. 18400731 10.1093/ajcn/87.4.1053

[B9] D’Amelio M. Cavallucci V. Cecconi F. (2010). Neuronal caspase-3 signaling: not only cell death. *Cell Death Differ.* 17 1104–1114. 10.1038/cdd.2009.180 19960023

[B10] Del Regno M. Adesso S. Popolo A. Quaroni A. Autore G. Severino L. (2015). Nivalenol induces oxidative stress and increases deoxynivalenol pro-oxidant effect in intestinal epithelial cells. *Toxicol. Appl. Pharmacol.* 285 118–127. 10.1016/j.taap.2015.04.002 25882925

[B11] Di Micco L. Marzocco S. Sirico M. L. Torraca S. Di Iorio B. (2012). Does daily dialysis improve hypertension in chronic haemodialysis patients? *Curr. Hypertens. Rev.* 8 291–295. 10.2174/1573402111208040291

[B12] Dou L. Jourde-Chiche N. Faure V. Cerini C. Berland Y. Dignat-George F. (2007). The uremic solute indoxyl sulfate induces oxidative stress in endothelial cells. *J. Thromb Haemost.* 5 1302–1308. 10.1111/j.1538-7836.2007.02540.x 17403109

[B13] Erol A. (2010). Are paradoxical cell cycle activities in neurons and glia related to the metabolic theory of Alzheimer’s disease? *J. Alzheimers Dis.* 19 129–135. 10.3233/JAD-2010-1211 20061632

[B14] Fann D. Y. Lee S. Y. Manzanero S. Tang S. C. Gelderblom M. Chunduri P. (2013). Intravenous immunoglobulin suppresses NLRP1 and NLRP3 inflammasome-mediated neuronal death in ischemic stroke. *Cell Death Dis.* 4:e790 10.1038/cddis.2013.326 PMC3789184 24008734

[B15] Faraonio R. Vergara P. Di Marzo D. Pierantoni M. G. Napolitano M. Russo T. (2006). p53 suppresses the Nrf2-dependent transcription of antioxidant response genes. *J. Biol. Chem.* 281 39776–39784. 10.1074/jbc.M605707200 17077087

[B16] Fortuño A. Beloqui O. San José G Moreno M. U. Zalba G. Díez J. (2005). Increased phagocytic nicotinamide adenine dinucleotide phosphate oxidase-dependent superoxide production in patients with early chronic kidney disease. *Kidney Int. Suppl.* 68(Suppl. 99) S71–S75. 10.1111/j.1523-1755.2005.09913.x 16336581

[B17] Frank-Cannon T. C. Alto L. T. McAlpine F. E. Tansey M. G. (2009). Does neuroinflammation fan the flame in neurodegenerative diseases? *Mol. Neurodegener.* 16 4–47. 10.1186/1750-1326-4-47 PMC2784760 19917131

[B18] Gelderblom M. Weymar A. Bernreuther C. Velden J. Arunachalam P. Steinbach K. (2012). Neutralization of the IL-17 axis diminishes neutrophil invasion and protects from ischemic stroke. *Blood* 120 3793–3802. 10.1182/blood-2012-02-412726 22976954

[B19] Guo J. T. Yu J. Grass D. de Beer F. C. Kindy M. S. (2002). Inflammation-dependent cerebral deposition of serum amyloid a protein in a mouse model of amyloidosis. *J. Neurosci.* 22 5900–5909. 12122052 10.1523/JNEUROSCI.22-14-05900.2002 PMC6757908

[B20] Heneka M. T. Carson M. J. El Khoury J. Landreth G. E. Brosseron F. Feinstein D. L. (2015). Neuroinflammation in Alzheimer’s disease. *Lancet Neurol.* 14 388–405. 10.1016/S1474-4422(15)70016-5 25792098 PMC5909703

[B21] Hosoya K. Tachikawa M. (2011). Roles of organic anion/cation transporters at the blood-brain and blood-cerebrospinal fluid barriers involving uremic toxins. *Clin. Exp. Nephrol.* 15 478–485. 10.1007/s10157-011-0460-y 21611756

[B22] Hsieh H. L. Yang C. M. (2013). Role of redox signaling in neuroinflammation and neurodegenerative diseases. *Biomed. Res. Int.* 2013:484613 10.1155/2013/484613 PMC3884773 24455696

[B23] Ichii O. Otsuka-Kanazawa S. Nakamura T. Ueno M. Kon Y. Chen W. Rosenberg A. Z. (2014). Podocyte injury caused by indoxyl sulfate, a uremic toxin and aryl-hydrocarbon receptor ligand. *PLoS ONE* 9:e108448 10.1371/journal.pone.0108448 PMC4171541 25244654

[B24] Kansanen E. Kuosmanen S. M. Leinonen H. Levonen A. L. (2013). The Keap1-Nrf2 pathway: mechanisms of activation and dysregulation in cancer. *Redox Biol.* 1 45–49. 10.1016/j.redox.2012.10.001 24024136 PMC3757665

[B25] Kensler T. W. Wajajayashi N. Biswal S. (2007). Cell survival responses to environmental sresses via Keap1-Nrf2-ARE pathway. *Annu. Rev. Pharmacol. Toxicol.* 47 89–116. 10.1146/annurev.pharmtox.46.120604.141046 16968214

[B26] Kim S. Y. Yang J. H. (2005). Neurotoxic effects of 2,3,7,8-tetrachlorodibenzo-p-dioxin in cerebellar granule cells. *Exp. Mol. Med.* 37 58–64. 10.1038/emm.2005.8 15761253

[B27] Kimmel P. L. Thamer M. Richard C. M. (1998). Psychiatric illness in patients with end-stage renal disease. *Am. J. Med.* 105 214–221. 10.1016/S0002-9343(98)00245-9 9753024

[B28] Kingwell K. (2012). Neurodegenerative disease: microglia in early disease stages. *Nat. Rev. Neurol.* 8:475 10.1038/nrneurol.2012.172 22890215

[B29] Krishnan A. V. Kiernan M. C. (2009). Neurological complications of chronic kidney disease. *Nat. Rev. Neurol.* 5 542–551. 10.1038/nrneurol.2009.138 19724248

[B30] Libetta C. Sepe V. Esposito P. Galli F. Dal Canton A. (2011). Oxidative stress and inflammation: implications in uremia and hemodialysis. *Clin. Biochem.* 44 1189–1198. 10.1016/j.clinbiochem.2011.06.988 21777574

[B31] Lin C. H. Juan S. H. Wang C. Y. Sun Y. Y. Chou C. M. Chang S. F. (2008). Neuronal activity enhances aryl hydrocarbon receptor-mediated gene expression and dioxin neurotoxicity in cortical neurons. *J. Neurochem.* 104 1415–1429. 10.1111/j.1471-4159.2007.05098.x 17973980

[B32] Liu S. Liu Y. Hao W. Wolf L. Kiliaan A. J. Penke B. (2012). TLR2 is a primary receptor for Alzheimer’s amyloid β peptide to trigger neuroinflammatory activation. *J. Immunol.* 188 1098–1107. 10.4049/jimmunol.1101121 22198949

[B33] Marchetti B. (1997). Cross-talk signals in the CNS: role of neurotrophic and hormonal factors, adhesion molecules and intercellular signaling agents in luteinizing hormone-releasing hormone (LHRH)-astroglial interactive network. *Front. Biosci.* 2 d88–d125. 10.2741/a177 9159216

[B34] Marinelli C. Di Liddo R. Facci L. Bertalot T. Conconi M. T. Zusso M. (2015). Ligand engagement of Toll-like receptors regulates their expression in cortical microglia and astrocytes. *J. Neuroinflammation* 30:244 10.1186/s12974-015-0458-6 PMC4696218 26714634

[B35] Marzocco S. Calabrone L. Adesso S. Larocca M. Franceschelli S. Autore G. (2015). Anti-inflammatory activity of horseradish (*Armoracia rusticana*) root extracts in LPS-stimulated macrophages. *Food Funct.* 6 3778–3788. 10.1039/c5fo00475f 26411988

[B36] Marzocco S. Dal Piaz F. Di Micco L. Torraca S. Sirico M. L. Tartaglia D. (2013). Very low protein diet reduces indoxyl sulfate levels in chronic kidney disease. *Blood Purif.* 35 196–201. 10.1159/000346628 23485887

[B37] Marzocco S. Popolo A. Bianco G. Pinto A. Autore G. (2010). Pro-apoptotic effect of methylguanidine on hydrogen peroxide-treated rat glioma cell line. *Neurochem. Int.* 57 518–524. 10.1016/j.neuint.2010.06.016 20599452

[B38] Morena M. Cristol J. P. Senécal L. Leray-Moragues H. Krieter D. Canaud B. (2002). Oxidative stress in hemodialysis patients: is NADPH oxidase complex the culprit? *Kidney Int.* 61 109–114. 10.1046/j.1523-1755.61.s80.20.x 11982824

[B39] Mozar A. Louvet L. Morlie‘re P. Godin C. Boudot C. Kamel S. (2011). Uremic toxin indoxyl sulfate inhibits human vascular smooth muscle cell proliferation. *Ther. Apher. Dial.* 15 135–139. 10.1111/j.1744-9987.2010.00885.x 21426504

[B40] Murray A. M. Knopman D. S. (2010). Cognitive impairment in CKD: no longer an occult burden. *Am. J. Kidney Dis.* 56 615–618. 10.1053/j.ajkd.2010.08.003 20851318 PMC2943494

[B41] Niwa T. (2010). Indoxyl sulfate is a nephro-vascular toxin. *J. Ren. Nutr.* 20 S2–S6. 10.1053/j.jrn.2010.05.002 20797565

[B42] Parker K. K. Norenberg M. D. Vernadakis A. (1980). “Transdifferentiation” of C6 glial cells in culture. *Science* 208 179–181. 10.1126/science.6102413 6102413

[B43] Pepe G. Sommella E. Manfra M. De Nisco M. Tenore G. C. Scopa A. (2015). Evaluation of anti-inflammatory activity and fast UHPLC-DAD-IT-TOF profiling of polyphenolic compounds extracted from green lettuce (*Lactuca sativa* L.; var. Maravilla de Verano). *Food Chem.* 167 153–161. 10.1016/j.foodchem.2014.06.105 25148972

[B44] Petersén A. Mani K. Brundin P. (1999). Recent advances on the pathogenesis of Huntington’s disease. *Exp. Neurol.* 157 1–18. 10.1006/exnr.1998.7006 10222105

[B45] Popolo A. Autore G. Pinto A. Marzocco S. (2013). Oxidative stress in cardiovascular and renal disease. *Free Radic. Res.* 47 346–356. 10.3109/10715762.2013.779373 23438723

[B46] Quincozes-Santos A. Nardin P. de Souza D. F. Gelain D. P. Moreira J. C. Latini A. (2009). The janus face of resveratrol in astroglial cells. *Neurotox. Res.* 16 30–41. 10.1007/s12640-009-9042-0 19526296

[B47] Radic J. Ljutic D. Radic M. Kovacic V. Sain M. Curkovic K. D. (2010). The possible impact of dialysis modality on cognitive function in chronic dialysis patients. *Neth. J. Med.* 68 153–157. 20421655

[B48] Raff A. C. Meyer T. W. Hostetter T. H. (2008). New insights into uremic toxicity. *Curr. Opin. Nephrol. Hypertens.* 17 560–565. 10.1097/MNH.0b013e32830f45b6 18941347

[B49] Richardson J. S. Subbarao K. V. Ang L. C. (1990). Biochemical indices of peroxidation in Alzheimer’s and control brains. *Trans. Am. Soc. Neurochem.* 21:113 10.1016/j.ejphar.2011.03.026

[B50] Rodríguez J. J. Olabarria M. Chvatal A. Verkhratsky A. (2009). Astroglia in dementia and Alzheimer’s disease. *Cell Death Differ.* 16 378–385. 10.1038/cdd.2008.172 19057621

[B51] Roger V. Lloyd P. M. H. Ronald P. M. (1997). The origin of the hydroxyl radical oxygen in the Fenton reaction. *Free Radic. Biol. Med.* 22 885–888. 10.1016/S0891-5849(96)00432-7 9119257

[B52] Schroeder J. C. Dinatale B. C. Murray I. A. Flaveny C. A. Liu Q. Laurenzana E. M. (2010). The uremic toxin 3-indoxyl sulfate is a potent endogenous agonist for the human aryl hydrocarbon receptor. *Biochemistry* 49 393–400. 10.1021/bi901786x 20000589 PMC2805781

[B53] Sehgal A. R. Grey S. F. De Oreo P. B. Whitehouse P. J. (1997). Prevalence, recognition, and implications of mental impairment among hemodialysis patients. *Am. J.Kidney Dis.* 30 41–49. 10.1016/S0272-6386(97)90563-1 9214400

[B54] Seliger S. L. Siscovick D. S. Stehman-Breen C. O. Gillen D. L. Fitzpatrick A. Bleyer A. (2004). Moderate renal impairment and risk of dementia among older adults: the cardiovascular health cognition study. *J. Am. Soc. Nephrol.* 15 1904–1911. 10.1097/01.ASN.0000131529.60019.FA 15213280

[B55] Shimizu H. Yisireyili M. Higashiyama Y. Nishijima F. Niwa T. (2013). Indoxyl sulfate upregulates renal expression of ICAM-1 via production of ROS and activation of NF-kB and p53 in proximal tubular cells. *Life Sci.* 92 143–148. 10.1016/j.lfs.2012.11.012 23201429

[B56] Smith M. A. Richey P. L. Taneda S. Kutty R. K. Sayre L. M. Monnier V. M. (1994). Advanced Maillard reaction end products, free radicals, and protein oxidation in Alzheimer’s disease. *Ann. N.Y. Acad. Sci.* 738 447–454. 10.1111/j.1749-6632.1994.tb21836.x 7832455

[B57] Tremblay M. È. Stevens B. Sierra A. Wake H. Bessis A. Nimmerjahn A. (2011). The role of microglia in the healthy brain. *J. Neurosci.* 31 16064–16069. 10.1523/JNEUROSCI.4158-11.2011 22072657 PMC6633221

[B58] Vanholder R. Argiles A. Baurmeister U. Brunet P. Clark W. Cohen G. (2001). Uremic toxicity: present state of the art. *Int. J. Artif. Organs* 24 695–725. 11817319

[B59] Vanholder R. De Smet R Glorieux G. Argilés A. Baurmeister U. Brunet P. (2003). Review on uremic toxins: classification, concentration, and interindividual variability. *Kidney Int.* 63 1934–1943. 10.1046/j.1523-1755.2003.00924.x 12675874

[B60] Varley J. Brooks D. J. Edison P. (2015). Imaging neuroinflammation in Alzheimer’s disease and other dementias: recent advances and future directions. *Alzheimers Dement.* 11 1110–1120. 10.1016/j.jalz.2014.08.105 25449529

[B61] Vernadakis A. (1996). Glia-neuron intercommunications and synaptic plasticity. *Prog. Neurobiol.* 49 185–214. 10.1016/S0301-0082(96)00012-3 8878303

[B62] Wang F. Zhang L. Wang Liu L. (2010). Level of kidney function correlates with cognitive decline. *Am. J. Nephrol.* 32 117–121. 10.1159/000315618 20714128

[B63] Watanabe I. Tatebe J. Namba S. Koizumi M. Yamazaki J. Morita T. (2013). Activation of aryl hydrocarbon receptor mediates indoxyl sulfate-induced monocyte chemoattractant protein-1 expression in human umbilical vein endothelial cells. *Circ. J* 77 224–230. 10.1253/circj.CJ-12-0647 23037589

[B64] Watanabe K. Watanabe T. Nakayama M. (2014). Cerebro–renal interactions: impact of uremic toxins on cognitive function. *Neurotoxicology* 44 184–193. 10.1016/j.neuro.2014.06.014 25003961

[B65] Williamson M. A. Gasiewicz T. A. Opanashuk L. A. (2005). Aryl hydrocarbon receptor expression and activity in cerebellar granule neuroblasts: implications for development and dioxin neurotoxicity. *Toxicol. Sci.* 83 340–348. 10.1093/toxsci/kfi031 15537747

[B66] Yu M. Kim Y. J. Kang D. H. (2011a). Indoxyl sulfate-induced endothelial dysfunction in patients with chronic kidney disease via an induction of oxidative stress. *Clin. J. Am. Soc. Nephrol.* 6 30–39. 10.2215/CJN.05340610 20876676 PMC3022246

[B67] Yu M. Li H. Liu Q. Liu F. Tang L. Li C. (2011b). Nuclear factor p65 interacts with Keap1 to repress the Nrf2-ARE pathway. *Cell Signal.* 23 883–892. 10.1016/j.cellsig.2011.01.014 21262351

[B68] Zhu W. Stevens A. P. Dettmer K. Gottfried E. Hoves S. Kreutz M. (2011). Quantitative profiling of tryptophan metabolites in serum, urine, and cell culture supernatants by liquid chromatography-tandem mass spectrometry. *Anal. Bioanal. Chem.* 401 3249–3261. 10.1007/s00216-011-5436-y 21983980

